# Multilevel Factors Associated with Frailty among the Rural Elderly in Korea Based on the Ecological Model

**DOI:** 10.3390/ijerph18084146

**Published:** 2021-04-14

**Authors:** Ah Ram Jang, Ju Young Yoon

**Affiliations:** 1 Center for Human-Caring Nurse Leaders for the Future by Brain Korea 21 (BK 21) Four Project, College of Nursing, Seoul National University, Seoul 03080, Korea; lillyforever@snu.ac.kr; 2 College of Nursing and Research Institute of Nursing Science, Seoul National University, Seoul 03080, Korea

**Keywords:** frailty, multilevel factors, rural elderly, ecological model, multilevel analysis

## Abstract

Frailty is prevalent in the rural elderly and, as a result, they are vulnerable to serious health problems. The purpose of this study was to examine the multilevel factors affecting frailty among the rural elderly using the ecological model. A total of 386 participants aged 65 years or older from 60 rural areas were included in the study. Frailty was measured using the Cardiovascular Health Study frailty index. Multilevel logistic regression analysis was used to identify the factors affecting frailty among the rural elderly. The results show that the levels of prevalence for robust, pre-frailty, and frailty groups were 81 (21%), 216 (56%), and 89 (23%), respectively. As for intrapersonal factors, old age, lower than middle school education, low and moderate levels of physical activity, depressive symptoms, and cognitive dysfunction significantly increased the risk of frailty; however, no interpersonal and community factors were significant in affecting frailty. The findings indicate that individualized strategies to encourage physical activity, prevent depressive symptoms, and preserve cognitive function are needed to prevent frailty in the rural elderly.

## 1. Introduction

As the elderly population increases, the prevalence of frailty is also steadily increasing. Frailty is defined as a condition of vulnerability to endogenous and exogenous stressors, resulting in negative health consequences, such as mortality, institutionalization, falls, and hospitalization [1,2,3,4]. Frailty is also related to social problems, such as the high medical cost of treating diseases in the elderly population [5] and the burden on families and society of taking care of the elderly [6]. It is therefore necessary to reduce frailty in the elderly population.

Factors affecting frailty have been widely investigated but research has mainly focused on individual factors, such as sociodemographic, physical, and mental aspects. In terms of sociodemographic aspects, increasing age, being female, and having a lower education and income increase the risk of frailty [7,8]. In terms of physical aspects, having three or more chronic diseases and malnutrition have both been found to be associated with frailty in older adults [8,9]. In terms of mental aspects, depression and anxiety have been found to be risk factors for frailty [10], and cognitive decline has also been found to be strongly associated with frailty [11].

According to the ecological model, several factors influence health at multiple levels, including intrapersonal, interpersonal, institutional, community, and public policy factors [12]. In accordance with this ecological perspective, some researchers have investigated the relationship between frailty and interpersonal factors and found there was a significant relationship. An 11-year longitudinal study in European countries showed that women with a poor social network have a higher risk of becoming frail [13]. In addition, social frailty, such as lack of social networking and going out, was a risk factor for developing frailty in the four years of a cohort study [14]. With regard to social support, a randomized control trial conducted among the community-dwelling elderly revealed that it has a positive effect on improving frailty [15]. Active participation in social activities and a high sense of socially belonging also seem to reduce frailty [16,17]. However, knowledge about the effects of interpersonal factors on frailty, including social networks and social support, is inconclusive due to numerous non-significant results [17,18], so these relationships need to be better established through follow-up studies.

With regard to community factors, some previous studies have found that frailty and community factors are indeed related. A poorly perceived neighborhood environment, including the absence of destinations and feeling unsafe at night, was associated with frailty in the urban elderly, whereas limited accessibility to aesthetic and recreational facilities were related to frailty in the rural elderly in community-dwelling older adults [19]. Feeling secure within the community was also a protective factor of frailty [16]. Concerning neighborhood characteristics, ethnic density and neighborhood socioeconomic deprivation were related to frailty [20,21]. Although studies have shown that community factors are related to frailty, few studies have focused on the relationship between the two. In addition, most existing studies have been conducted on an individual level, with very few studies encompassing intrapersonal, interpersonal, and community factors using an ecological model.

To date, research on frailty among the elderly in rural areas has been largely neglected. In general, rural areas have a higher prevalence of frailty than urban areas [22], and the elderly in rural areas are considered particularly vulnerable to serious health problems caused by frailty. Despite the vulnerability of this group, few studies have focused on frailty in the rural elderly population, particularly on the relationship between frailty and multilevel factors. Since rural areas have different social environmental characteristics from urban areas, a different perspective and approach are needed. Therefore, the purpose of this study was to explore the multilevel factors affecting frailty in the rural elderly based on the ecological model.

## 2. Materials and Methods

### 2.1. Study Participants and Data Collection

We conducted a survey from May to September 2020 on the elderly aged 65 years or older residing in rural areas in the Chungcheong regions (Daejeon, Sejong, and Chungnam) of South Korea. Due to the limited accessibility of rural areas, the survey was conducted in cooperation with primary health care posts (PHCPs) located in rural areas. PHCPs are part of the national health care system of South Korea—which was created in 1980—and deliver primary health care, including medical treatment and prescription of medications, to rural residents [23]. Each PHCP serves about 500–1000 rural residents [24] and is managed by a community health practitioner (CHP). 

In multilevel analysis, the main limitation is about the sample size at the highest level [25]. In order to increase the power of the analysis, it is important that the number of samples at the group level (level 2) is large, not the average cluster size [26]. To prevent biased estimates of the second-level standard errors, at least 50 samples at level 2 are required [25]. Based on this evidence, and considering dropouts, we focused on 60 rural areas and recruited as many participants as possible in each area.

Using a convenience sampling method, the principal investigator (PI) contacted PHCPs and requested permission to contact residents for the study. A total of 60 PHCPs located across the 60 rural areas agreed to cooperate with this study (Daejeon: 8 PHCPs, Sejong: 2 PHCPs, Chungnam: 50 PHCPs). Data collection was conducted by the PI and surveyors who had experience as community health nurses, and one-day training was undertaken to help in understanding questionnaire items. For data collection, the PI and surveyors visited the PHCPs to explain the purpose and method of the study to residents and screened the residents for eligibility after receiving voluntary informed consent in person. When participants met the inclusion criteria, they filled out the questionnaire themselves, and the surveyors measured the frailty of participants. The inclusion criteria for participants in the present study were as follows: (1) aged 65 years or older; (2) without serious cognitive impairment (K-MMSE > 17); (3) ambulatory with or without an assistive device; and (4) able to provide informed consent. A total of 408 participants from 60 rural areas agreed to participate in the study, but 22 participants were excluded from the analysis due to an incomplete frailty index. Therefore, data from 386 participants in 60 rural areas were used for the final analysis. Ethical approval for the study was obtained from Seoul National University (IRB No. 2005/001-004). 

### 2.2. Dependent Variable: Frailty

The frailty of participants was measured using the Cardiovascular Health Study (CHS) frailty index. The CHS frailty index includes five components: unintended weight loss, poor grip strength, exhaustion, reduced walking speed, and low physical activity level [7]. For unintended weight loss, participants were asked whether they had experienced unintentional weight loss greater than 4.5 kg in the preceding year; those who answered yes received 1 point. Grip strength was measured twice for each hand with a hand dynamometer (Camry-EH101, Henqi, Guangdong, China); the highest value was used in the analysis. One point was given for grip strength of less than 26 kg for males and 18 kg for females [27]. Exhaustion was assessed by asking for participants’ responses to two statements from the Center for Epidemiological Studies Depression (CES-D) scale: “I felt that everything I did was an effort” and “I could not get going”. One point was given if a participant answered “yes, three or more days in a week” to either of these questions [28]. Reduced walking speed was averaged by walking 4 m twice at a usual gait speed, and 1 point was given for less than <0.8 m/s [27]. Physical activity levels were measured using the International Physical Activity Questionnaire (IPAQ) by asking participants about the amount of physical activity they had undertaken over the previous week. One point was given for values below 494.6 kcal for males and 283.5 kcal for females, which corresponded to the lowest 20% of total energy consumed by gender [29]. Participants who had 3 points or more were classified as frail, those with 1–2 points were classified as pre-frail, and those with 0 points were classified as robust. In this study, multilevel analysis was conducted by dividing frailty status into a dichotomous score; not frail (0–2 points) and frail (3 points or more).

### 2.3. Independent Variables: Multilevel Factors

**Figure 1:**
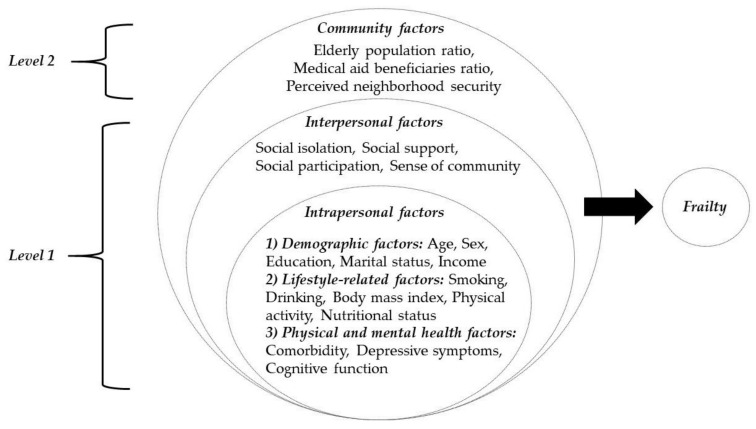
The conceptual framework of this study.

Multilevel factors were used as independent variables, including intrapersonal, interpersonal, and community factors, based on the ecological model [12]. Studies from the literature suggest that intrapersonal factors (e.g., demographic, lifestyle-related, and physical/mental health factors), interpersonal factors (e.g., social network, social support, social participation, and social cohesion), and community factors (e.g., subjective neighborhood experience and neighborhood characteristics) are associated with frailty [17,30]. Therefore, intrapersonal, interpersonal, and community factors were selected for the study and classified into two levels according to the level of data collection. Intrapersonal and interpersonal factors were level 1 factors because they were answered at the individual level, and community factors were level 2 factors because they were collected at the local level. The framework of this study is shown in Figure 1. 

#### 2.3.1. Intrapersonal Factors

Demographic factors were investigated, including age, sex, the highest level of education completed, marital status, and household yearly income. Lifestyle-related factors were also assessed, such as smoking, drinking, body mass index (BMI), physical activity, and nutritional status. To classify current smokers and drinkers, the average daily smoking amount and the average number of drinking days per month were surveyed, respectively. BMI was calculated by weight (kg) ÷ height^2^ (m^2^). Physical activity was measured using the IPAQ [31], which calculates metabolic equivalent task (MET) min per week, indicating the total energy expenditure during physical activity. Physical activity was classified into three categories according to the MET min per week level of physical activity: high (at least 3000 MET minutes/week), moderate (at least 600 MET minutes/week), and low (<600 MET minutes/week). Nutritional status was measured using the Mini Nutritional Assessment–Short Form (MNA-SF) [32]. The MNA-SF consists of six items: decrease in food intake, weight loss, mental stress, mobility, neuropsychiatric problems, and BMI. A total score of 12–14 points was classified as normal, and a score of 0–11 points was classified as malnutrition. Physical and mental health factors included comorbidity, depressive symptoms, cognitive function, and quality of life. Participants were also asked whether they had been diagnosed with a chronic disease by a physician, including hypertension, diabetes, cancer, chronic pulmonary diseases, liver diseases, cardiovascular diseases, cerebrovascular diseases, mental diseases, arthritis, and prostate diseases. Comorbidity was defined as having two or more chronic diseases. Depressive symptoms were measured using the Geriatric Depression Scale Short Form Korea version (GDSSF-K), validated for the Korean elderly [33]. The range of total scores was 0–15, with a higher score indicating severe depressive symptoms. Cognitive function was measured using the Korean version of the Mini-Mental State Examination (K-MMSE), with a range of total scores from 0 to 30 points [34]. Participants with less than 24 points were classified as having cognitive dysfunction. 

#### 2.3.2. Interpersonal Factors

According to the ecological model, interpersonal factors indicate formal and informal social networks and social support from others [12]. Based on this definition and previous studies [13,14,16,17], social isolation, social support, social participation, and sense of community were selected as interpersonal factors. Social isolation was assessed by the Korean version of the Lubben Social Network Scale–6 (LSNS-6) [35], including three questions on interactions with family and relatives and three questions on interactions with neighbors and friends. Total scores ranged from 0 to 30 points, and less than 12 points indicated a risk of social isolation. Social support was evaluated using the Enhancing Recovery in Coronary Heart Disease (ENRICHD) Social Support Instrument (ESSI), with a total score from 0 to 6 [36]. A score of 6 or more was classified as high social support, and a score of 5 or less was classified as average or low social support. Social participation in religious activities, volunteer activities, social group activities, leisure or cultural activities, and political activities was assessed with a five-point Likert scale [37]. The total score was distributed from the lowest score of 5 points to the highest score of 25 points, and a higher score indicated active participation in social activities. Sense of community was assessed using the sense of community scale with 15 questions, including questions on satisfaction, solidarity, belonging and mutual influence, and emotional intimacy, answered using five-point Likert scales [38]. Total scores were calculated by averaging the 15 questions, with high scores indicating a high sense of community. 

#### 2.3.3. Community Factors

Community factors refer to mediating structures, indicating the relationships among networks within defined boundaries [12]. Community factors included subjective neighborhood experience (perceived neighborhood security) and neighborhood characteristics (the ratio of elderly in the rural population and that of medical aid beneficiaries). Perceived neighborhood security was associated with frailty in a previous study [16]. In another study, neighborhood socioeconomic deprivation was related to frailty [21]; thus, the ratio of elderly in the rural population and that of medical aid beneficiaries were chosen for community factors. These factors, especially the ratio of the elderly population, are useful in determining the economic characteristics of a region, as they serve as components of the regional deprivation index determining the economic level of the region [39]. 

Perceived neighborhood security was assessed for four items(quiet and peaceful; spacious and roomy; safe; orderly with good public security) on a four-point Likert scale [40]. The range of total scores was 4–16 points, with higher total scores indicating a more secure neighborhood. To identify the perceived safety score at the regional level, the scores for each rural area were generated by aggregating individual responses within each area. Using the community health post information system obtained from PHCPs, the total population, the number of elderly (aged 65 years or older), and the number of medical aid beneficiaries in each rural area were identified. The ratio for the elderly was calculated by dividing the number of elderly individuals by the total population and multiplying it by 100. The ratio for medical aid beneficiaries was calculated by dividing the number of medical aid beneficiaries by total population and multiplying it by 100. High ratios for the elderly population and the medical aid beneficiaries indicated that there were many elderly people and medical aid beneficiaries in the community. 

### 2.4. Data Analysis

For descriptive statistical analysis, the study participants were divided into three groups: frail, pre-frail, and robust. ANOVA, for continuous variables, and chi-square tests, for the categorical variables, were performed to compare whether there were differences in general characteristics for each group. Statistical analysis was performed using SPSS version 25.0 (IBM Corp., Armonk, NY, USA).

To identify multilevel factors affecting frailty, a random intercept multilevel logistic regression model was used for analysis by dividing participants into two groups; frail and not frail (pre-frail and robust). Since individuals (level 1) were nested within neighborhoods (level 2), multilevel analysis was adopted as an appropriate statistical method to handle hierarchical data. For analysis, the logit link function was used, which is appropriate with binary data (frail vs. not frail). The logit link function is shown below in Equation (1), where $\eta_{ij}$ is the logit value of the *i-th* individual in the *j-th* neighborhood. $\psi_{ij}$ is the probability that the *i-th* individual in the *j-th* neighborhood has a frail status (Equation (2)).

(1) $$\eta_{ij}=\;\log\left(\frac{\psi_{ij}}{1-\psi_{ij}}\right)$$

(2) $$\psi_{ij}=P\left(Y_{ij\;}=1\right)$$

A random intercept multilevel logistic regression model with single explanatory variables at the individual and community levels is described by Equations (3) and (4). In Equation (3), $X_{ij}$ is the explanatory variable of the *i-th* individual, $\beta_{0j}$ is the intercept, and $\beta_{1j}$ is the effect of the explanatory variable $X_{ij}$ on $\eta_{ij}$. $\beta_{0j}$ in level 1 consisted of components from level 2, including the intercept $\gamma_{00}$, the explanatory variable $W_{j}$ of the *j-th* neighborhood, and the error term $u_{0j}$ (Equation (4)).

(3) $$\begin{array}{ll} \mathrm{Level}\;1\;(\mathrm{individual})\text{:} & \eta_{ij}=\beta_{0j}+\beta_{1j}X_{ij} \end{array}$$

(4) $$\begin{array}{ll} \;\mathrm{Level}\;2\;(\mathrm{community})\text{:} & \beta_{0j}=\gamma_{00}+\gamma_{01}W_{j}+u_{0j},\;u_{0j}\sim N\left(0,\sigma_{u}^{2}\right) \end{array}$$

$$\begin{array}{ll} & \;\beta_{1j}=\gamma_{10}, \end{array}$$

The random intercept multilevel logistic regression model combining the independent variables of level 1 and level 2 is described below (Equation (5)).

(5) $$\eta_{ij}=\gamma_{00}+\gamma_{01}W_{j}+\gamma_{10}X_{ij}+u_{0j},\;u_{0j}\sim N\left(0,\sigma_{u}^{2}\right)$$

The detailed procedure for the multilevel analysis was as follows. First, the intra-class correlation coefficient (ICC) was calculated from a null model with no explanatory variables inputted. The ICC refers to the ratio of the between-group variance to the total variance. The formula for calculating the ICC is described in Equation (6).

(6) $$\mathrm{ICC}\;=\frac{\hat{\sigma_{\delta }^{2}}}{\hat{\sigma_{\delta }^{2}}+\pi^{2}/3}$$

In this study, the ICC was 0.146, indicating that about 14.6% of the total variance of frailty was explained by the effect at the community level (level 2). This indicated a significant difference in frailty between neighborhoods, so it was necessary to apply a multilevel analysis. Thus, to identify multilevel factors affecting frailty at each level, a multilevel analysis was conducted by creating three models. In model 1, intrapersonal and interpersonal variables (level 1) were added to the null model. Then, in model 2, only community variables (level 2) were included in the null model. Finally, in model 3, we added both intrapersonal and interpersonal variables (level 1) and community variables (level 2) into the model. We used random intercept multilevel logistic regression by fixing the slopes of all independent variables. To facilitate the interpretation of the intercepts, we used group mean centering for level 1 continuous variables and grand mean centering for level 2 continuous variables. As a result of the analysis, the odds ratio (OR) and 95% confidence interval (CI) values were calculated. Lastly, the −2 log likelihood (-2LL) and Akaike information criterion (AIC) were obtained to determine the goodness of fit of the model. Mplus software (version 8.5) was used for multilevel analysis and the significance level was set at *p* < 0.05.

## 3. Results

### 3.1. General Characteristics of the Study Participants

Of the 386 respondents, the number of participants in the robust, pre-frail, and frail categories were 81 (21%), 216 (56%), and 89 (23%), respectively (Table 1). For intrapersonal factors, the mean age of the frail elderly (79.79 years) was much higher than that of the robust (73.44 years) and pre-frail (75.94 years) elderly. The frail elderly were more likely to be female; less educated; single, divorced, or widowed; and earning a lower yearly income. They also had lower drinking rates, lower BMI, higher rates of low-intensity exercise, and higher rates of malnutrition than the non-frail elderly. In addition, a high proportion of the frail elderly had comorbidity, severe depressive symptoms, and cognitive dysfunction. For interpersonal factors, the frail elderly suffered more social isolation and received less social support than the non-frail elderly. In terms of social participation and having a sense of community, the frail elderly had lower scores than the non-frail elderly.

### 3.2. Multilevel Factors Affecting Frailty in the Rural Elderly

Multilevel logistic regression analysis was used to identify the factors affecting frailty among the rural elderly. The results of the multilevel logistic regression model are presented in Table 2. With only intrapersonal and interpersonal factors (level 1) included in the model (model 1), increased age (OR = 1.209, 95% CI 1.044–1.402), lower than middle school education (OR = 1.358, 95% CI 1.061–1.738), moderate (OR = 1.302, 95% CI 1.047–1.621) and low (OR = 1.665, 95% CI 1.342–2.067) levels of physical activity, malnutrition (OR = 1.154, 95% CI 1.010–1.318), and cognitive dysfunction (OR = 1.154, 95% CI 1.047–1.271) increased the risk of frailty; however, there were no significant interpersonal factors associated with frailty. In model 2, including only community factors, there were no significant associations between community factors and frailty. In the final model (model 3), intrapersonal, interpersonal, and community factors were all added. Among the level 1 intrapersonal factors, increased age was associated with increased risk of frailty (OR = 1.220, 95% CI 1.050–1.418). In addition, those with lower than middle school education had greater odds of frailty compared to those with an education equal to or more advanced than middle school (OR = 1.297, 95% CI 1.020–1.649). Furthermore, compared to those with high levels of physical activity, those with low (OR = 1.728, 95% CI 1.370–2.177) and moderate (OR = 1.357, 95% CI 1.069–1.721) levels of physical activity were more likely to be frail. High levels of depressive symptoms were also related to frailty (OR = 1.130, 95% CI 1.003–1.273). Lastly, older adults who suffered from cognitive dysfunction were more likely to become frail than those without cognitive dysfunction (OR = 1.166, 95% CI 1.055–1.289). No significant factors affected frailty in level 1 interpersonal and level 2 community factors.

## 4. Discussion

Given the dearth of studies about the factors related to frailty among the rural elderly, this study examined the prevalence of frailty and multilevel factors that affect frailty based on the ecological model. Among the factors for the two levels, including individual and environmental factors, only individual-level variables, particularly intrapersonal factors, were statistically associated with frailty. 

The present study also investigated the prevalence of frailty among the rural elderly using CHS frailty criteria. About 23% of the participants were frail and 56% had pre-frail status, which indicates that there was a high prevalence of frailty among the rural elderly. In a systematic review analyzing 21 cohort studies, the prevalence of frailty among community-dwelling older adults ranged widely from 4.0% to 59.1%; specifically, the weighted prevalence was 9.9% for frail status and 44.2% for pre-frail status when the results were limited to studies using CHS frailty criteria [41]. This study was only conducted on the rural elderly, which resulted in a higher prevalence of frailty than in previous studies [41]. In general, frailty is more prevalent in rural than urban populations [22] due to characteristics specific to rural areas such as the old age of inhabitants, low socio-economic status, and limited accessibility of health care services [42,43]. Prevalence rates of frailty in this study are similar to those in a previous study that reported 17.4% for frail status and 52.6% for pre-frail status in the Korean rural elderly [44].

In terms of demographic factors, an increase in age was associated with frailty, which corresponds well with results from earlier studies [45,46]. According to a systematic review, the prevalence of frailty steadily increases with age [41]. In addition, almost all people appear to be frail by the age of 95 due to health deficits [45]; thus, strategies to reduce frailty are needed, especially for older people. In addition, having an education level lower than middle school was also related to frailty, which is consistent with the results of previous studies [43,47]. Although education level has no direct impact on the pathophysiology of frailty, it can affect the lifestyle of individuals, which may be closely related to the progression of frailty [30]. 

Among lifestyle-related factors, low and moderate levels of physical activity were associated with frailty compared to a high level of physical activity. This finding agrees well with many previous studies [22,48,49]. For example, in a 12-month longitudinal cohort study, low physical activity increased the likelihood of frailty and decreased the likelihood of improving frailty [48]. Furthermore, low levels of physical activity combined with sedentary behavior have been found to increase the likelihood of frailty by 2.83 times [50]. Over the 10 years of the ensuing follow-up study, compared with the sedentary behavior, moderate and vigorous physical activity significantly reduced the likelihood of frailty in older adults [51]. As physical activity and frailty may be closely related, encouraging physical activity and preventing an inactive lifestyle is necessary to reduce frailty among the rural elderly.

In this study, malnutrition was significantly associated with frailty in model 1 and marginally associated with frailty in model 3. A meta-analysis including 10 studies with a total of 5447 older adults has revealed that malnutrition and physical frailty are related [52]. In fact, malnutrition is a key factor related to frailty since it affects all frailty criteria (i.e., unintentional weight loss, low muscle strength, feeling of exhaustion, reduced physical activity capacity, and slow walking speed) [53]. In addition, insufficient dietary intake causes the reduction of protein synthesis and loss of muscle mass, leading to weakness [53]. A healthy dietary pattern, such as having a diet high in protein, fruit, and vegetables, could be helpful to protect from frailty [54,55].

In terms of mental health factors, depressive symptoms and cognitive dysfunction were associated with frailty. Depression and frailty are related to each other; moreover, they seem to have a reciprocal interaction [56]. According to a meta-analysis, people with depression have a fourfold increased risk of becoming frail and people with frailty also have a fourfold higher risk of becoming depressed [56]. With regard to cognitive function, the results of the present study are quite similar to earlier studies [11,57,58]. In a systematic review with a meta-analysis, a strong association between cognitive decline and physical frailty in the elderly over 60 years old was found [11]. This association may be attributed to the mechanisms that underlie frailty and cognitive decline, including common risk factors such as chronic disease, poor cardiovascular health, inflammation, and hormonal imbalance [58]. Furthermore, cognitive dysfunction can contribute to the occurrence of frailty through behavioral changes that limit physical activity and harm nutrition [58]. 

There was no significant association between interpersonal factors and frailty in this study. In a systematic review analyzing 15 studies, the relationship between frailty and social networks or social support was found to be unclear, which supports our findings [17]. However, there were several studies showing a significant relationship between social factors and frailty. In a longitudinal cohort study in England with 2817 older adults, social isolation increased the risk of becoming frail only in men [59]. A pathway study undertaken on Korean immigrants in the USA found that social support directly and negatively influences frailty [60]. In addition, social participation, such as helping others or participating in religious activities, has been found to be related to reductions of frailty [61]. Social vulnerability in terms of engagement, including lack of participation in group, religious, and physical leisure activities, have also been found to increase frailty [18]. In these previous studies, frailty was measured with the Rockwood Frailty Scale [62] or the Korean version of the FRAIL scale [63], whereas we measured frailty using the CHS frailty index. Since the results pertaining to the relationship between frailty and social factors were different depending on which frailty assessment tool was used [59], the differences in the results among the studies may be due to inconsistencies between instruments for measuring frailty. In addition, we conducted the survey during the coronavirus disease (COVID-19) pandemic, when many people were forced into home confinement and social distancing, resulting in fewer social activities [64]. Although rural populations were less affected by the social restrictions, some residents may have been affected, which could have hidden the relationship between frailty and social participation in this study. Further research is needed after the COVID-19 pandemic restrictions.

To determine whether regional characteristics affect individual frailty, perceived neighborhood security and the ratio of the elderly population and that of medical aid beneficiaries (i.e., indicating the deprivation of the region) were used as community factors in this study. In the multilevel analysis, there was no statistically significant relationship between community factors and frailty, and these findings are similar to those of an earlier study on the rural elderly [19,65]. Seo [19] suggested that there was no relationship between perceived neighborhood safety and frailty among the rural elderly. The study by Manrique-Espinoza [65] used a deprivation index, including education level, household environment, and work-related income, and it did indicate regional vulnerability, but it was not related to frailty. In contrast, another study in which the majority of participants were from urban areas found a linear relationship between frailty and neighborhood socioeconomic deprivation [21]. Although those studies used community factors that were different from those used in this study, it seems that the relationship between community factors and frailty is different in urban and rural areas. To prevent frailty among the rural elderly, we recommend an intervention focusing on individual-level variables, more specifically intrapersonal factors, rather than community-level variables. 

This study has some limitations. First, the cross-sectional study design could not be used to infer causality. Second, this study should be interpreted with caution given its small sample size. Third, the study might exhibit selection bias due to the use of a convenience sampling method and the inclusion only of participants who were ambulatory. Lastly, this survey was conducted during the COVID-19 pandemic; therefore, interpersonal factors, such as social participation, and community-level factors could have been underestimated. However, to the best of our knowledge, this is the first study to comprehensively analyze the relationship between frailty and individual and environmental factors in the rural elderly. The present study contributes to a comprehensive understanding of the multilevel factors influencing frailty in the rural elderly, and provides new insights into how to reduce frailty in the rural population. In the future, longitudinal research with sufficient data should be conducted to determine the causal relationship between frailty and multilevel factors in the rural elderly. In addition, based on the results of this study, an intervention study to reduce frailty in the rural elderly is needed, focusing on individual-level variables. Although this study was conducted in the Daejeon, Sejong, and Chungnam-do regions of South Korea, the results have relevant implications for other developed countries and Asian regions with similar cultures to Korea. 

## 5. Conclusions

Our study is the first study to adopt an ecological model to identify multilevel factors affecting frailty among the rural elderly. This study shows that frailty is highly prevalent in the rural elderly population. Individual factors, especially intrapersonal factors such as old age, lower than middle school education, low and moderate levels of physical activity, depressive symptoms, and cognitive dysfunction, were associated with frailty. Therefore, policymakers should primarily consider health policies to reduce frailty among the rural elderly. In addition, to prevent frailty among the rural elderly, we recommend an intervention focusing on individual-level variables, more specifically intrapersonal factors, rather than community-level variables. Health educators and practitioners need to develop an individual-focused intervention plan to encourage physical activity, prevent depression, and preserve cognitive function to reduce frailty in the rural elderly. 

## Figures and Tables

**Table 1:** General characteristics of participants based on frailty status (*n* = 386).

Variables	Mean ± SD ^1^ or *n* (%)				*p*
	Overall (*n* = 386)	Robust (*n* = 81)	Pre-Frail (*n* = 216)	Frail (*n* = 89)	
**Intrapersonal factors**					
**Age (years)**	76.31 ± 6.22	73.44 ± 5.75	75.94 ± 5.91	79.79 ± 5.80	<0.001
**Sex**					
Male	127 (32.9)	31 (38.3)	79 (36.6)	17 (19.1)	0.007
Female	259 (67.1)	50 (61.7)	137 (63.4)	72 (80.9)	
**Education**					
≥Middle school	86 (22.5)	32 (40.5)	52 (24.3)	2 (2.2)	<0.001
<Middle school	296 (77.5)	47 (59.5)	162 (75.7)	87 (97.8)	
**Marital status**					
Married	233 (60.4)	54 (66.7)	136 (63.0)	43 (48.3)	0.025
Single/divorced/widowed	153 (39.6)	27 (33.3)	80 (37.0)	46 (51.7)	
**Income**					
≥20 million won	109 (28.5)	32 (40.0)	63 (29.3)	14 (15.9)	0.002
<20 million won	274 (71.5)	48 (60.0)	152 (70.7)	74 (84.1)	
**Smoking**					
Currently non-smoker	364 (94.3)	77 (95.1)	200 (92.6)	87 (97.8)	0.199
Currently smoker	22 (5.7)	4 (4.9)	16 (7.4)	2 (2.2)	
**Drinking**					
No	312 (80.8)	58 (71.6)	174 (80.6)	80 (89.9)	0.010
Yes	74 (19.2)	23 (28.4)	42 (19.4)	9 (10.1)	
**BMI ^2^ (kg/m^2^)**	24.45 ± 3.62	25.11 ± 2.79	24.44 ± 3.68	23.87 ± 4.05	0.080
**Physical activity**					
High level	74 (19.2)	22 (27.2)	49 (22.7)	3 (3.4)	<0.001
Moderate level	153 (39.6)	44 (54.3)	88 (40.7)	21 (23.6)	
Low level	159 (41.2)	15 (18.5)	79 (36.6)	65 (73.0)	
**Nutritional status**					
Normal	261 (67.8)	71 (87.7)	150 (69.8)	40 (44.9)	<0.001
Malnutrition	124 (32.2)	10 (12.3)	65 (30.2)	49 (55.1)	
**Comorbidity**					
No	169 (43.8)	44 (54.3)	102 (47.2)	23 (25.8)	<0.001
Yes	217 (56.2)	37 (45.7)	114 (52.8)	66 (74.2)	
**Depressive symptoms (0–15)**	4.79 ± 4.00	2.70 ± 2.79	4.35 ± 3.52	7.80 ± 4.36	<0.001
**Cognitive function**					
Normal	311 (80.8)	71 (88.8)	182 (84.3)	58 (65.2)	<0.001
Cognitive dysfunction	74 (19.2)	9 (11.3)	34 (15.7)	31 (34.8)	
**Interpersonal factors**					
**Social isolation**					
No	275 (71.6)	69 (85.2)	150 (70.1)	56 (62.9)	0.004
Yes	109 (28.4)	12 (14.8)	64 (29.9)	33 (37.1)	
**Social support**					
High	300 (77.9)	70 (86.4)	169 (78.6)	61 (68.5)	0.018
Average/low	85 (22.1)	11 (13.6)	46 (21.4)	28 (31.5)	
**Social participation (5–25)**	9.82 ± 3.91	11.44 ± 4.07	9.87 ± 3.93	8.21 ± 2.98	<0.001
**Sense of community (1–5)**	3.88 ± 0.63	4.09 ± 0.57	3.88 ± 0.62	3.71 ± 0.66	<0.001
**Community factors**					
**Perceived neighborhood security (4–16)**	3.34 ± 0.52	3.33 ± 0.52	3.37 ± 0.49	3.29 ± 0.60	0.513
**Elderly population ratio (%)**	44.10 ± 13.32	45.48 ± 14.39	44.28 ± 13.14	42.46 ± 12.75	0.328
**Medical aid beneficiaries ratio (%)**	3.06 ± 2.92	2.88 ± 2.66	3.20 ± 3.17	2.86 ± 2.49	0.547

^1^ SD: standard deviation; ^2^ BMI: body mass index. Missing data were excluded from the analysis.

**Table 2:** Factors associated with frailty among the rural elderly using multilevel logistic regression (*n* = 386).

Variables		Model 1		Model 2		Model 3	
Fixed Effects		OR ^1^	(95% CI ^2^)	OR^1^	(95% CI ^2^)	OR ^1^	(95% CI ^2^)
**Level 1** **Intrapersonal Factors**	**Sex**						
	Male	1				1	
	Female	1.005	(0.875–1.156)			0.997	(0.867–1.147)
	**Age (years)**	1.209	(1.044–1.402)			1.220	(1.050–1.418)
	**Education**						
	≥Middle school	1				1	
	<Middle school	1.358	(1.061–1.738)			1.297	(1.020–1.649)
	**Marital status**						
	Married	1				1	
	Single/divorced/widowed	0.960	(0.845–1.898)			0.962	(0.845–1.095)
	**Income**						
	≥20 million won	1				1	
	<20 million won	1.033	(0.886–1.204)			1.022	(0.878–1.190)
	**Smoking**						
	Currently non-smoker	1				1	
	Currently smoker	0.909	(0.789–1.047)			0.917	(0.799–1.053)
	**Drinking**						
	No	1				1	
	Yes	0.919	(0.789–1.047)			0.920	(0.786–1.078)
	**BMI ^3^ (kg/m^2^)**						
	**Physical activity**						
	High level	1				1	
	Moderate level	1.302	(1.047–1.621)			1.357	(1.069–1.721)
	Low level	1.665	(1.342–2.067)			1.728	(1.370–2.177)
	**Nutritional status**						
	Normal	1				1	
	Malnutrition	1.154	(1.010–1.318)			1.153	(0.999–1.331)
	**Comorbidity**						
	No	1				1	
	Yes	1.038	(0.910–1.182)			1.037	(0.908–1.183)
	**Depressive symptoms (0–15)**	1.116	(0.989–1.260)			1.130	(1.003–1.273)
	**Cognitive function**						
	Normal	1				1	
	Cognitive dysfunction	1.154	(1.047–1.271)			1.166	(1.055–1.289)
**Interpersonal Factors**	**Social isolation**						
	No	1				1	
	Yes	0.991	(0.881–1.116)			0.976	(0.865–1.103)
	**Social support**						
	High	1				1	
	Average/low	1.097	(0.969–1.242)			1.105	(0.968–1.264)
	**Social participation (5–25)**	0.891	(0.788–1.008)			0.890	(0.789–1.006)
	**Sense of community** **(1–5)**	0.953	(0.842–1.079)				
**Level 2** **Community** **Factors**	**Perceived neighborhood security (4–16)**			1.065	(0.721–1.571)	0.855	(0.566–1.292)
	**Elderly population ratio (%)**			0.775	(0.537–1.119)	0.812	(0.558–1.181)
	**Medical aid beneficiaries ratio (%)**			0.877	(0.624–1.232)	0.807	(0.555–1.172)
**Random Effects**							
**Community-Level Variance (SE ^4^)**		0.955 (0.692)		0.456 (0.292)		0.831 (0.618)	
**Model Fit**							
**-2LL ^5^**		247.480		398.744		239.906	
**AIC ^6^**		287.481		408.744		285.906	

^1^ OR: Odds Ratio; ^2^ CI: Confidence Interval; ^3^ BMI: Body Mass Index; ^4^ SE: Standard Error; ^5^ LL: Log Likelihood; ^6^ AIC: Akaike Information Criterion. Data from 24, 11, 34 participants in models 1, 2, and 3, respectively, were excluded from the analysis due to incomplete data.

## Data Availability

The datasets analyzed during the current study are not publicly available but are available from the corresponding author on reasonable request.

## References

[1] Rockwood K., Stadnyk K., MacKnight C., McDowell I., Hébert R., Hogan D.B. (1999). A brief clinical instrument to classify frailty in elderly people. Lancet.

[2] Speechley M., Tinetti M. (1991). Falls and Injuries in Frail and Vigorous Community Elderly Persons. J. Am. Geriatr. Soc..

[3] Winograd C.H. (1991). Targeting strategies: An overview of criteria and outcomes. J. Am. Geriatr. Soc..

[4] Morley J.E., Vellas B., Van Kan G.A., Anker S.D., Bauer J.M., Bernabei R., Cesari M., Chumlea W., Doehner W., Evans J. (2013). Frailty Consensus: A Call to Action. J. Am. Med. Dir. Assoc..

[5] Robinson T.N., Wu D.S., Stiegmann G.V., Moss M. (2011). Frailty predicts increased hospital and six-month healthcare cost following colorectal surgery in older adults. Am. J. Surg..

[6] Covinsky K.E., Eng C., Lui L.-Y., Sands L.P., Sehgal A.R., Walter L.C., Wieland D., Eleazer G.P., Yaffe K. (2001). Reduced Employment in Caregivers of Frail Elders: Impact of Ethnicity, Patient Clinical Characteristics, and Caregiver Characteristics. J. Gerontol. Ser. A Boil. Sci. Med. Sci..

[7] Fried L.P., Tangen C.M., Walston J., Newman A.B., Hirsch C., Gottdiener J., Seeman T., Tracy R., Kop W.J., Burke G. (2001). Frailty in Older Adults: Evidence for a Phenotype. J. Gerontol. Ser. A Biol. Sci. Med. Sci..

[8] He B., Ma Y., Wang C., Jiang M., Geng C., Chang X., Ma B., Han L. (2019). Prevalence and Risk Factors for Frailty Among Community-Dwelling Older People in China: A Systematic Review and Meta-Analysis. J. Nutr. Health Aging.

[9] Kim J., Lee Y., Won C.W., Lee K.E., Chon D. (2018). Nutritional Status and Frailty in Community-Dwelling Older Korean Adults: The Korean Frailty and Aging Cohort Study. J. Nutr. Health Aging.

[10] Zhao W., Zhang Y., Liu X., Yue J., Hou L., Xia X., Zuo Z., Liu Y., Jia S., Dong B. (2020). Comorbid depressive and anxiety symptoms and frailty among older adults: Findings from the West China health and aging trend study. J. Affect. Disord..

[11] Furtado G.E., Caldo A., Rieping T., Filaire E., Hogervorst E., Teixeira A.M.B., Ferreira J.P. (2018). Physical frailty and cognitive status over-60 age populations: A systematic review with meta-analysis. Arch. Gerontol. Geriatr..

[12] McLeroy K.R., Bibeau D., Steckler A., Glanz K. (1988). An Ecological Perspective on Health Promotion Programs. Health Educ. Q..

[13] Haider S., Grabovac I., Drgac D., Mogg C., Oberndorfer M., Dorner T.E. (2019). Impact of physical activity, protein intake and social network and their combination on the development of frailty. Eur. J. Public Health.

[14] Makizako H., Shimada H., Doi T., Tsutsumimoto K., Hotta R., Nakakubo S., Makino K., Lee S. (2018). Social Frailty Leads to the Development of Physical Frailty among Physically Non-Frail Adults: A Four-Year Follow-Up Longitudinal Cohort Study. Int. J. Environ. Res. Public Health.

[15] Luger E., Dorner T.E., Haider S., Kapan A., Lackinger C., Schindler K. (2016). Effects of a Home-Based and Volunteer-Administered Physical Training, Nutritional, and Social Support Program on Malnutrition and Frailty in Older Persons: A Randomized Controlled Trial. J. Am. Med. Dir. Assoc..

[16] Cramm J.M., Nieboer A.P. (2013). Relationships between frailty, neighborhood security, social cohesion and sense of belonging among community-dwelling older people. Geriatr. Gerontol. Int..

[17] Duppen D., Van der Elst M.C., Dury S., Lambotte D., De Donder L. (2019). D-SCOPE The social environment’s relationship with frailty: Evidence from existing studies. J. Appl. Gerontol..

[18] Andrew M.K., Keefe J.M. (2014). Social vulnerability from a social ecology perspective: A cohort study of older adults from the National Population Health Survey of Canada. BMC Geriatr..

[19] Seo Y., Kim M., Shim H., Won C.W. (2021). Differences in the Association of Neighborhood Environment with Physical Frailty Between Urban and Rural Older Adults: The Korean Frailty and Aging Cohort Study (KFACS). J. Am. Med. Dir. Assoc..

[20] Aranda M.P., Ray L.A., Snih S.A., Ottenbacher K.J., Markides K.S. (2011). The protective effect of neighborhood composition on increasing frailty among older Mexican Americans: A barrio advantage?. J. Aging Health.

[21] Lang I.A., Hubbard R.E., Andrew M.K., Llewellyn D.J., Melzer D., Rockwood K. (2009). Neighborhood Deprivation, Individual Socioeconomic Status, and Frailty in Older Adults. J. Am. Geriatr. Soc..

[22] Yu R., Wu W.-C., Leung J., Hu S.C., Woo J. (2017). Frailty and Its Contributory Factors in Older Adults: A Comparison of Two Asian Regions (Hong Kong and Taiwan). Int. J. Environ. Res. Public Health.

[23] Lee C.Y., Cho Y.H. (2012). Evaluation of a Community Health Practitioner Self-care Program for Rural Korean Patients with Osteoarthritis. J. Korean Acad. Nurs..

[24] Kim W.J., Yoon S.N., June K.J., Kim S.Y., Kim C.M., Kim H.S. (2010). Community Health Nursing.

[25] Maas C.J., Hox J.J. (2005). Sufficient sample sizes for multilevel modeling. Methodology.

[26] Snijders T.A. (2005). Power and sample size in multilevel linear models. Encycl. Stat. Behav. Sci..

[27] Chen L.-K., Liu L.-K., Woo J., Assantachai P., Auyeung T.-W., Bahyah K.S., Chou M.-Y., Hsu P.-S., Krairit O., Lee J.S. (2014). Sarcopenia in Asia: Consensus Report of the Asian Working Group for Sarcopenia. J. Am. Med. Dir. Assoc..

[28] Orme J.G., Reis J., Herz E.J. (1986). Factorial and discriminant validity of the Center for Epidemiological Studies Depression (CES-D) scale. J. Clin. Psychol..

[29] Son J., Kim S., Won C., Choi H., Kim B., Park M. (2015). Physical frailty predicts medical expenses in community-dwelling, elderly patients: Three-year prospective findings from living profiles of older people surveys in Korea. Eur. Geriatr. Med..

[30] Mello A.D.C., Engstrom E.M., Alves L.C. (2014). Health-related and socio-demographic factors associated with frailty in the elderly: A systematic literature review. Cad. Saúde Pública.

[31] Oh J.Y., Yang Y.J., Kim B.S., Kang J.H. (2007). Validity and Reliability of Korean Version of International Physical Activity Questionnaire (IPAQ) Short Form. J. Korean Acad. Fam. Med..

[32] Kaiser M.J., Bauer J.M., Ramsch C., Uter W., Guigoz Y., Cederholm T., Thomas D.R., Anthony P., Charlton K.E., MNA-International Group (2009). Validation of the Mini Nutritional Assessment short-form (MNA^®^-SF): A practical tool for identification of nutritional status. J. Nutr. Health Aging.

[33] Kee B.S. (1996). A Preliminary Study for the Standardization of Geriatric Depression Scale Short Form-Korea Version. J. Korean Neuropsychiatr Assoc..

[34] Kang Y.W., Na D.L., Hahn S.H. (1997). A validity study on the korean mini-mental state examination (K-MMSE) in dementia patients. J. Korean Neurol. Assoc..

[35] Hong M., Casado B.L., Harrington D. (2011). Validation of Korean Versions of the Lubben Social Network Scales in Korean Americans. Clin. Gerontol..

[36] Mitchell P.H., Powell L., Blumenthal J., Norten J., Ironson G., Pitula C.R., Froelicher E.S., Czajkowski S., Youngblood M., Huber M. (2003). A short social support measure for patients recovering from myocardial infarction: The ENRICHD Social Support Inventory. J. Cardiopulm. Rehabil..

[37] Cheon E.-Y. (2010). Correlation of Social Network Types on Health Status of Korean Elders. J. Korean Acad. Nurs..

[38] Kim K.J., Kim S.S. (1998). A Study on the Residents’ Sense of Community in Korea. J. Korean Community Dev. Soc..

[39] Yun J.-W., Kim Y.-J., Son M. (2016). Regional Deprivation Index and Socioeconomic Inequalities Related to Infant Deaths in Korea. J. Korean Med. Sci..

[40] Yang M.-J., Shih C.-H., Kawachi I. (2002). Development and validation of an instrument to measure perceived neighbourhood quality in Taiwan. J. Epidemiol. Community Health.

[41] Collard R.M., Boter H., Schoevers R.A., Voshaar R.C.O. (2012). Prevalence of Frailty in Community-Dwelling Older Persons: A Systematic Review. J. Am. Geriatr. Soc..

[42] Boulos C., Salameh P., Barberger-Gateau P. (2016). Malnutrition and frailty in community dwelling older adults living in a rural setting. Clin. Nutr..

[43] Szanton S.L., Seplaki C.L., Thorpe R.J., Allen J.K., Fried L.P. (2009). Socioeconomic status is associated with frailty: The Women’s Health and Aging Studies. J. Epidemiol. Community Health.

[44] Jung H.-W., Jang I.-Y., Lee Y.S., Lee C.K., Cho E.-I., Kang W.Y., Chae J.H., Lee E.J., Kim D.H. (2016). Prevalence of Frailty and Aging-Related Health Conditions in Older Koreans in Rural Communities: A Cross-Sectional Analysis of the Aging Study of Pyeongchang Rural Area. J. Korean Med. Sci..

[45] Song X., Mitnitski A., Rockwood K. (2010). Prevalence and 10-Year Outcomes of Frailty in Older Adults in Relation to Deficit Accumulation. J. Am. Geriatr. Soc..

[46] Ye B., Gao J., Fu H. (2018). Associations between lifestyle, physical and social environments and frailty among Chinese older people: A multilevel analysis. BMC Geriatr..

[47] Hoogendijk E.O., Heymans M.W., Deeg R.J., Huisman M. (2018). Socioeconomic Inequalities in Frailty among Older Adults: Results from a 10-Year Longitudinal Study in the Netherlands. Gerontology.

[48] Ahmad N.S., Hairi N.N., Said M.A., Kamaruzzaman S.B., Choo W.Y., Hairi F., Othman S., Ismail N., Peramalah D., Kandiben S. (2018). Prevalence, transitions and factors predicting transition between frailty states among rural community-dwelling older adults in Malaysia. PLoS ONE.

[49] Kendhapedi K.K., Devasenapathy N. (2019). Prevalence and factors associated with frailty among community-dwelling older people in rural Thanjavur district of South India: A cross-sectional study. BMJ Open.

[50] Da Silva V.D., Tribess S., Meneguci J., Sasaki J.E., Garcia-Meneguci C.A., Carneiro J.A.O., Virtuoso J.S. (2019). Association between frailty and the combination of physical activity level and sedentary behavior in older adults. BMC Public Health.

[51] Rogers N.T., Marshall A., Roberts C.H., Demakakos P., Steptoe A., Scholes S. (2017). Physical activity and trajectories of frailty among older adults: Evidence from the English Longitudinal Study of Ageing. PLoS ONE.

[52] Verlaan S., Ligthart-Melis G.C., Wijers S.L., Cederholm T., Maier A.B., de van der Schueren M.A. (2017). High Prevalence of Physical Frailty Among Community-Dwelling Malnourished Older Adults–A Systematic Review and Meta-Analysis. J. Am. Med. Dir. Assoc..

[53] Yannakoulia M., Ntanasi E., Anastasiou C.A., Scarmeas N. (2017). Frailty and nutrition: From epidemiological and clinical evidence to potential mechanisms. Metabolism.

[54] Pour Fard N.R., Amirabdollahian F., Haghighatdoost F. (2019). Dietary patterns and frailty: A systematic review and meta-analysis. Nutr. Rev..

[55] Coelho-Júnior H.J., Rodrigues B., Uchida M., Marzetti E. (2018). Low Protein Intake Is Associated with Frailty in Older Adults: A Systematic Review and Meta-Analysis of Observational Studies. Nutrients.

[56] Soysal P., Veronese N., Thompson T., Kahl K.G., Fernandes B.S., Prina A.M., Solmi M., Schofield P., Koyanagi A., Tseng P.-T. (2017). Relationship between depression and frailty in older adults: A systematic review and meta-analysis. Ageing Res. Rev..

[57] Feng Z., Lugtenberg M., Franse C., Fang X., Hu S., Jin C., Raat H. (2017). Risk factors and protective factors associated with incident or increase of frailty among community-dwelling older adults: A systematic review of longitudinal studies. PLoS ONE.

[58] Robertson D.A., Savva G.M., Kenny R.A. (2013). Frailty and cognitive impairment—A review of the evidence and causal mechanisms. Ageing Res. Rev..

[59] Gale C.R., Westbury L., Cooper C. (2018). Social isolation and loneliness as risk factors for the progression of frailty: The English Longitudinal Study of Ageing. Age Ageing.

[60] Sagong H., Yoon J. (2021). Pathways among Frailty, Health Literacy, Acculturation, and Social Support of Middle-Aged and Older Korean Immigrants in the USA. Int. J. Environ. Res. Public Health.

[61] Woo J., Goggins W., Sham A., Ho S.C. (2005). Social Determinants of Frailty. Gerontology.

[62] Rockwood K., Mitnitski A.B., Macknight C. (2002). Some mathematical models of frailty and their clinical implications. Rev. Clin. Gerontol..

[63] Jung H.-W., Yoo H.J., Park S.-Y., Kim S.-W., Choi J.Y., Yoon S.-J., Kim C.-H., Kim K.-I. (2016). The Korean version of the FRAIL scale: Clinical feasibility and validity of assessing the frailty status of Korean elderly. Korean J. Intern. Med..

[64] Ammar A., Chtourou H., Boukhris O., Trabelsi K., Masmoudi L., Brach M., Bouaziz B., Bentlage E., How D., Ahmed M. (2020). COVID-19 Home Confinement Negatively Impacts Social Participation and Life Satisfaction: A Worldwide Multicenter Study. Int. J. Environ. Res. Public Health.

[65] Manrique-Espinoza B., De Snyder N.S., Moreno-Tamayo K., Gutiérrez-Robledo L.M., Salinas-Rodríguez A., Avila-Funes J.A. (2015). Frailty and Social Vulnerability in Mexican Deprived and Rural Settings. J. Aging Health.

